# Quantum DFT studies on the drug delivery of favipiravir using pristine and functionalized chitosan nanoparticles

**DOI:** 10.1038/s41598-023-49298-5

**Published:** 2023-12-11

**Authors:** Sheyda Ataei, Ebrahim Nemati-Kande, Aidin Bahrami

**Affiliations:** Department of Physical Chemistry, Faculty of Chemistry, Urmia University, Urmia, Iran

**Keywords:** Computational chemistry, Density functional theory, Quantum chemistry

## Abstract

Considering the spread of the COVID-19 pandemic, finding new drugs along with the development of effective drug delivery methods can help in the treatment of this disease. For this reason, in this research work, the possibility of drug-delivery of Favipiravir (FP), one of the drugs approved in the treatment of COVID-19, by pristine chitosan (Chit) nanoparticles (NP), and functionalized chitosan nanoparticles with *N*-acylate, *N*-methyl, *O*-acetyl, and Oxazoline functional groups was studied using quantum mechanical DFT methods at B3LYP-D3(BJ)/6-311 + g(d,p) theoretical level in water medium. The QTAIM, NBO, DOS, frontier orbital, conceptual-DFT indices, and non-covalent interaction analysis were further implemented to investigate the possible interactions between FP and Chit NPs. The results show that the adsorption of FP on Chit NPs is done through the creation of hydrogen bonds, and the highest absorption energy of − 18.15 kcal/mol between pristine chitosan and FP. In the case of all functionalized Chit NPs, a decrease in the absorption energy is observed, which is more noticeable in the case of *N*-acylated and *O*-acetyl functionalize Chit NPs, and indicates the weakening of the van der Waals interactions for these cases. Considering the compatibility of Chit NPs with the human body and their non-toxicity, as well as the fact that factors such as pH, solubility, the ionic strength, and so on can be adjusted to control the release rate using the functionalized Chit NPs, it seems that the results of this work can be a comprehensive guide to design the drug delivery methods of FP drug using Chit NPs, to reduce the symptoms of COVID-19 disease.

## Introduction

The COVID-19 pandemic, caused by the novel coronavirus SARS-CoV-2, originated in December 2019 in Wuhan, Hubei Province, China. It quickly spread globally, leading to significant health and economic consequences. In December 2019, the first cases of a mysterious pneumonia-like illness are reported in Wuhan, and the world health organization (WHO) declares COVID-19 a pandemic due to its global spread, in December 2019^1^. As of September 2023, there have been over 770 million confirmed cases and more than 7 million deaths worldwide^2^. Now, vaccination has been done in many countries, but at the same time, the pandemic continues, with ongoing waves and new variants emerging.

Some drugs and treatments currently approved or recommended for the treatment of COVID-19 are Remdesivir^3^, Dexamethasone^4^, Tocilizumab^5^, Baricitinib^6^, Convalescent plasma therapy^7^, Bamlanivimab^8^, Casirivimab and Imdevimab^9^, Favipiravir^3^, Interferon beta-1a^10^, and Molnupiravir^11^. Favipiravir (FP), also known as Avigan, is an antiviral drug that has shown potential in treating COVID-19^3^. Favipiravir inhibits the replication of RNA viruses, including SARS-CoV-2, the virus that causes COVID-19^12,13^. It acts as a viral RNA polymerase inhibitor, preventing viral replication within human cells. Several studies have been conducted to evaluate the efficacy and safety of favipiravir in COVID-19 patients. These studies include randomized controlled trials and observational studies^14^. Results have been mixed, with some studies indicating positive outcomes, while others show limited benefits^15^. Favipiravir is generally considered as a potential treatment option for COVID-19 patients who are experiencing mild to moderate symptoms^16^. It may be more effective if administered during the early stages of the disease, before severe respiratory complications occur. Some studies have suggested that favipiravir can reduce the duration of symptoms, viral load, and hasten viral clearance in COVID-19 patients^17^. It may also help prevent disease progression and reduce the risk of severe complications. However, the overall impact on mortality rates is still under investigation. Favipiravir has also gained regulatory approvals for COVID-19 treatment in some countries, including Japan, Russia, and India^17^.

Along with conducting clinical studies on the effect of FP on patients, other studies at the molecular level to investigate the molecular mechanism of action, as well as extensive studies using computational methods to investigate the possibility of effective drug delivery of FP have also been conducted in the last few years^18–20^. The Al_12_N_12_ nanocages was suggested by Ibrahim et al.^21^ as a suitable nanocarrier of FP. The adsorption of FP on B_n_N_n_ nanocages with n = 12, 16, 20, and 24 has also been studied using the quantum mechanical density functional theory (DFT) methods^22^. Results confirmed that the binding energy of FP…BN nanocages as well as the recovery time of FP from nanocages were decreased by increasing the size of the nanocage, and therefore the size of the nanocages can act as a construable parameter in targeted drug delivery of FP using BN nanocages. In another study using DFT methods, Piya and coworkers^23^ found that pristine boron nitride nano sheets (BNNS) and especially BNNS doped with In atoms capable of carrying FP drug with the binding energy of − 2.4 eV in aqueous media. Mg_12_O_12_ and Zn_12_O_12_ nanocages has also been suggested as a potential nanocarriers of the FP through the DFT, molecular docking and molecular mechanics studies^24^. These authors also found that, binding affinity of these nanocages inside the SARS-CoV-2 main protease receptor is larger than that of FP. Pandey and coworkers^25^ modified the structure of FP and designed 12 new similar molecules, and found using the conceptual-DFT indices such as chemical potential (*μ*), hardness (*η*), softness (*S*), and electrophilicity (*ω*) along with the molecular docking methods that 4 of these new structures may be efficient in treatment of COVID-19. Although these valuable studies suggested new methods for targeted drug delivery using nanoparticles, the toxicity and compatibility as well as the side effects of these nanoparticles in the human-body are unknown and require more studies.

Chitosan is derived from the natural chitin polymer, either fully or partially deacetylated through treatment with strong alkalis^26^. The primary source of chitin is the shells of crustaceans, although it can also be found in renewable sources like insect exoskeletons and fungi^27^. Chitosan, as the most well-known biopolymer, has attracted a lot of attention from researchers in various fields, including agriculture, food industry, and health. Chitosan can protect biomolecules against harsh environmental conditions, including pH, temperature and light. On the other hand, chitosan has a major drawback that limits its biological applications, and that is its low solubility at pH above 6.5. This limitation arises from the robust network of both intermolecular and intramolecular hydrogen bonds present among the amino and hydroxyl groups. Additionally, factors like molecular weight, deacetylation-to-acetylation ratio, distribution of acetyl groups, pH, and the type of acid used play crucial roles in its solubility and ion concentration^28^. The *N*-acylate^29^, *N*-methyl^30^, *O*-acetyl^31^, and Oxazoline (Oxo)^32^ functionalized chitosan are some functionalized chitosan (FChit) derivatives that have properties optimized for specific applications, and thus can overcome the inherent limitations of pure chitosan.

Due to the good compatibility of chitosan with human-body and its acceptable level of toxicity, we decided to investigate the efficiency of chitosan and some functionalized chitosan nanoparticles in the adsorption and therefore drug-delivery of FP. DFT methods were performed at the theoretical level of B3LYP-D3(BJ)/6-311 + g(d,p), and also Bader’s topology analysis^33^, natural bond orbital (NBO) methods^34^, density of state (DOS) spectra, noncovalent interaction, molecular orbitals, and conceptual-DFT indices were implemented to obtain detailed information at atomistic level regarding the interaction of FP with Chit.

## Computational methods

All electronic calculations were done using Gaussian 16 software^35^, and using the B3LYP functional^35^. The Pople’s split-valence double-zeta 6-311 + g(d,p)^36–38^ basis set also used in all calculations. Currently, many functionals with different capabilities were introduced and used by researchers in different fields. The B3LYP^39,40^ functional is categorized as a hybrid exchange–correlation functional. It is created by merging the Becke 88^41^ exchange functional with the correlation functional of LYP, along with the local density approximation for the correlation functional^42^. B3LYP is among the most frequently employed functionals in chemistry for investigating typical chemical compounds. However, it, along with numerous other functionals, has a notable drawback, particularly in its ability to accurately estimate the weak, long-range London dispersion effects^43^. One of the approaches used to assess and address these effects involves employing the semi-empirical dispersion correction method (DFT-D) initially introduced by Grimme^43^. In one of the last modifications of this correction (D3(BJ))^44^, Becke-Johnson (BJ) dispersion correction method was used, which has led to a better approximation, especially in the mid-range and short-range interaction behavior of dispersion effects. Therefore, the D3(BJ) DFT-D correction has also been implemented in this work to consider the dispersion effects. Also, since most of the drugs must be dissolved in a suitable solvent, and since water is the main solvent of the body, the solvent effect of water was also considered using the polarizable continuum model (PCM)^45^.

At first, FP molecule and Chit nanoparticles as trimers were designed and optimized by B3LYP-D3(BJ)/6–311 + g(d,p) method at water environment. Then electrostatic potential energy surface (ESP) of these molecules were calculated at the same level of theory using the Multiwfn^46^ software and using the wavefunctions generated by Gaussian, and the ESP surface was analyzed and all local minima and maxima were found. The ESP surfaces were then visualized by VMD software^47^. Due to the complex structure of chitosan nanoparticles and the existence of many interaction-cites to interact with the FP, these ESP maps were used to design the Chit/FP complexes. In this way, the minimum points on the ESP surface of Chit were placed next to the maximum points on the ESP surface of FP and vice versa with a vertical distance of 2 Å between FP and Chit, in order to create the maximum amount of electrostatic attraction between them. In this way, 14 different combinations were created for Chit/FP complex. But in the case of functionalized chitosan (FChit) NPs, our goal is only to investigate the effect of the added functional group of Chit, and because the other parts of the functionalized Chit molecules are similar to pristine Chit, only FChit/FP complex were made from the functional group area of FChit. The constructed Chit/FP complexes were further optimized using B3LYP-D3(BJ)/6-311 + g(d,p) method at water environment, and the basis-set superposition error was corrected by the counterpoise correction method of Boys and Bernardi B^48^. The adsorption energy (*E*_*ad*_) of the FP on chitosan was estimated by:

1 $$E_{ad} = E_{Chit/FP} - E_{Chit} - E_{FP} + E_{BSSE}$$

where *E*_*Chit/FP*_, *E*_*Chit*_, and *E*_*FP*_ are the energies of the Chit/FP complex, the individual Chit, and FP, respectively. *E*_*BSSE*_ is also the BSSE correction to *E*_*ad*_. *E*_*ad*_ is actually made up of two other main parts, namely, the first is the interaction energy (*E*_*int*_) resulting from the interaction between Chito and FP, and normally stabilize the complex compared to the pristine components, while the second part of *E*_*ad*_ is related to the deformation energy (*E*_*def*_) of the geometric structure of the molecule, which mainly causes instability of the pristine molecules. Naturally, for a complex consisting of two Chit and FP molecule, *E*_*def*_ can be divided into two Chit $\left( {E_{def}^{Chit} } \right)$ and FP $\left( {E_{def}^{{{\text{FP}}}} } \right)$ parts. These energies can be calculated as,

2 $$E_{{{\text{bin}}}} = E_{{{\text{int}}}} + \,E_{{{\text{def}}}}$$

3 $$E_{int} = E_{{\text{Chit/FP}}} - \left( {E_{{\text{Chit - complex}}} + E_{{\text{FP - complex}}} } \right)$$

4 $$E_{def} = \,E_{def}^{Chit} + E_{def}^{{{\text{FP}}}} = \,\left( {E_{{\text{Chit - complex}}} - E_{{{\text{pristine}} - {\text{Chit}}}} } \right)\, + \,\,\left( {E_{{\text{FP - complex}}} - E_{{{\text{pristine}} - {\text{FP}}}} } \right)$$

In these equations, *E*_Chit-complex_ and *E*_FP-complex_ are the energies of the individual Chit and FP in the Chit/FP complex structure, respectively, which were obtained from the calculation of the energy for the isolated Chit or FP in the complex geometrical structure. Also, *E*_pristine*-*Chit_ and *E*_pristine*-*FP_ are the energies of initial optimized Chit and FP, respectively.

The highest occupied molecular orbital (HOMO) and the lowest unoccupied molecular orbital (LUMO) energies (ε) were also used to compute the conceptual-DFT indices of chemical potential (*μ*), hardness (*η*), softness (*S*), and electrophilicity (*ω*), using the formulas proposed by Janak^49^ and Parr et al.^50^ These conceptual DFT indices were widely used in the literature^51,52^, and can be calculated using

5 $$\mu = \left( {\frac{\partial \,E}{{\partial \,N}}} \right)_{{\upsilon (\vec{r})}} \cong \,\frac{{(\varepsilon_{L} + \varepsilon_{H} )}}{2}$$

6 $$\eta = \frac{1}{2}\left( {\frac{{\partial \,^{2} E}}{{\partial \,N^{2} }}} \right)_{{\upsilon (\vec{r})}} \cong \,\frac{{(\varepsilon_{L} - \varepsilon_{H} )}}{2}$$

7 $$S = 1/\eta$$

8 $$\omega = \frac{{\mu^{2} }}{2\eta }$$

Also, the iso-surfaces of HOMO and LUMO orbitals were calculated using the cubegen utility of Gaussian 16, and then visualized using the VMD software at iso-surfaces of 0.002 *a*.*u*.

The Bader’s theory^33,53^ which is based on the topological analysis of the electron density (ρ(r)), and known as quantum theory of atoms in molecules (QTAIM), can be used to obtain the bond critical points (BCP). The QTAIM analysis of the BCPs can also be used to investigate the possibility of strong chemical interactions (i.e., chemical bonds for electron densities greater than 0.1) or physical interactions (i.e., van der Waals interactions with electron densities typically less than 0.1). Natural bond orbital (NBO)^54^ analysis also provides a humble picture of interactions based on the simple concept of Lewis acid (i.e., donor of non-bonding electron pairs) and base (i.e., the acceptor), and so can be helpful in interpreting the results. Therefore, the QTAIM analysis was done using Multiwfn software^46^, and NBO analysis was performed by NBO 6 program^54^, to better interpret the results. The total electron density of states (TDOS) of the complex and projected density of states (PDOS) of FP and Chit in the complex were also computed and plotted based on the energies of the resulting molecular orbitals and using Gaussian broadening with the full width at half maximum parameter set to 0.1 eV as implemented in Multiwfn software. The reduced density gradient (RDG) plots of the Chit/FP complexes have also been calculated using Multiwfn software.

## Result and discussions

### Optimized structures

**Figure 1:**
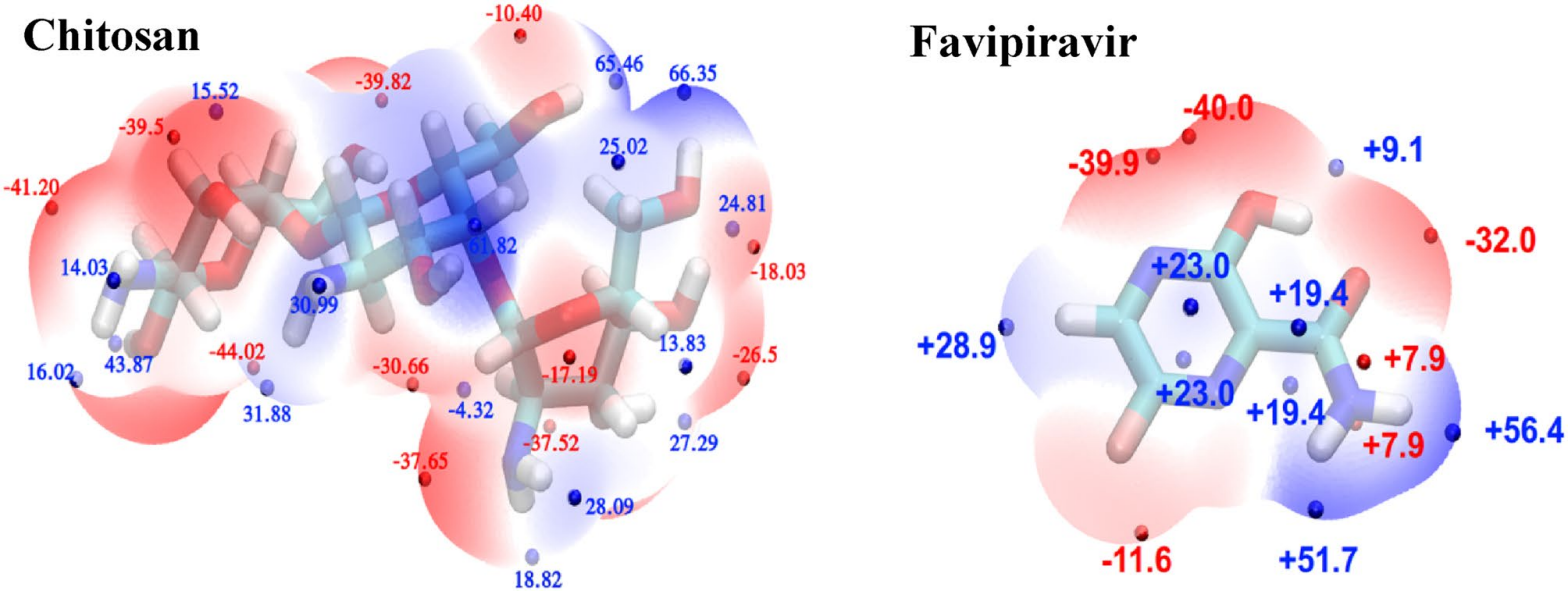
Electrostatic potential energy surface (ESP) of optimized Chitosan nanoparticles and Favipiravir molecule. The red and blue points represent the local minima and maxima points of the ESP surface, respectively.

**Figure 2:**
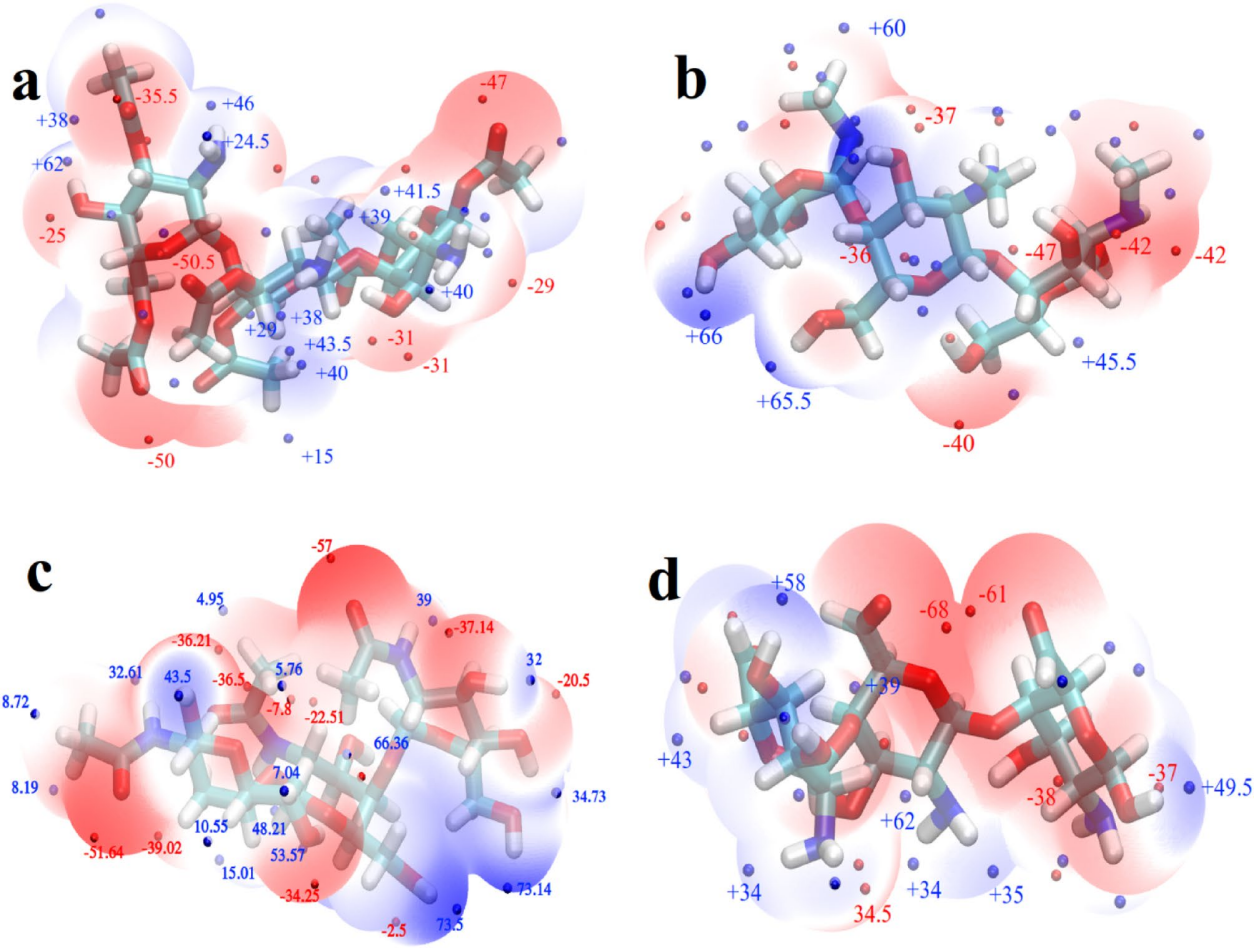
The electrostatic potential energy surface (ESP) for the optimized structures of (**a**) *O*-acetyl, (**b**) *N*-methyl, (**c**) *N*-acylate, and (**d**) Oxo- functionalized chitosan nanoparticles. The red and blue points represent the local minima and maxima points of the ESP surface, respectively.

In this work the potential application of Chit NPs for adsorption of FP drug were studied theoretically using the quantum DFT methods. Moreover, Chit has been functionalized with four different types of functional groups, i.e., *N*-acylate, *N*-methyl, *O*-acetyl, and Oxazoline (Oxo)-Chitosan, to investigate the effect of functionalization on the adsorption of FP. It is critical to highlight that, the structure of a polymer and its spatial configuration in a real experimental condition depend on many factors such as the number of monomers, pH, ionic strength of the environment, temperature, solvent type, inter- and intra-molecular interactions such as hydrogen bonding, and so on. It is practically impossible to simulate such conditions, especially using quantum methods. However, the simplest choice to investigate the interaction between drug and polymer is to limit the study to the interaction between a monomer and a drug molecule. However, in this work, Chit monomer was replaced by a trimer in order to consider, at least, the effect of neighboring monomers on such interaction. Although, Chit is actually polymerized longer than the trimer, and the contact conformation between Chit and FP is more restricted, the calculations were limited to trimers only for the convenience of the calculation. The structure of FP, Chit, and functionalized Chitosan (FChit) trimers were optimized by B3LYP functional with 6-311 + g(d,p) basis set in water as solvent and considering the D3(BJ) dispersion correction. The optimized molecular structure can be seen in Figs. 1 and 2.

Due to the complex geometric structure of the studied polymers, there are many adsorption-cites on Chit for FP. In such complex situations, the electrostatic potential energy (ESP) surface can be used to validate the most probable states. In other words, by calculating the ESP and taking into account the physical absorption of FP on Chit, we expect that the maximum absorption energy will occur in a situation where positions with positive potential energy of FP are located in the vicinity of more negative potential energies of Chit and vice versa. Therefore, the ESP of the FP, Chit, and FChit NPs were calculated at theoretical level applied for the optimization and using the algorithm provided in Multiwfn software, and the local minima and maxima were found, and the calculated ESPs have also been shown in Figs. 1 and 2.

These figures represent the charge distribution of the molecule, indicating its properties and how it interacts with other constituent molecules. Electrophilic reactivity is represented by red color, indicating negative regions of the molecule that experience stronger repulsion and are located on electronegative atoms rich in electrons. Positive regions (blue color) correspond to nucleophilic reactivity and indicate stronger attraction, suggesting a lack of electrons.

**Figure 3:**
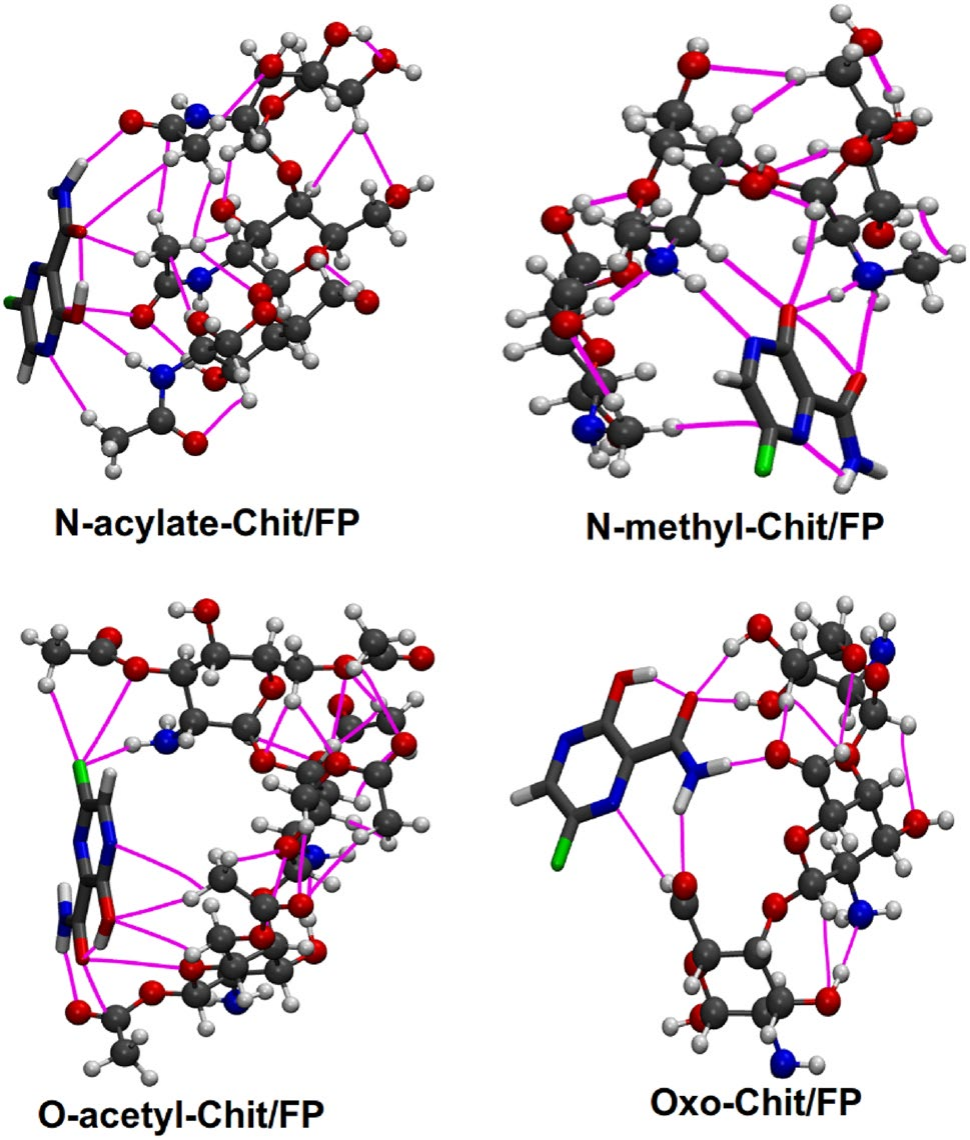
Optimized structure of the functionalized chitosan nanoparticles and Favipiravir complexes, along with the bond-paths (magenta lines) representing the possible bonding interactions. The bond paths were calculated from the Bader’s QTAIM analysis.

As seen from Fig. 1, for FP molecule, the highest potential energy with a value of + 56.4 kcal/mol is located around the NH_2_ group, which makes this position prone to nucleophilic attack. Also, the global minimum point with a value of − 40.0 kcal/mol is located in the vicinity of OH group connected to the ring, and therefore this area is the most susceptible position for electrophilic attack. Based on the calculated ESP surface, 14 different positions with the highest electrostatic attraction energy for the placement of FP next to Chit were obtained. In all 14 different positions the FP molecule was located with the distance of 2 Å next to the Chitosan nanoparticle. All of these initial structure were also optimized by B3LYP functional with 6-311 + g(d,p) basis set in water solvent and considering the D3(BJ) dispersion correction. The optimized structures were shown in Figure S1 of supplementary information. In the case of functionalized chitosan nanoparticles, since the objective here is to examine how the adsorption of FP is influenced by the functional group. To achieve this, the FP molecule was positioned in close proximity to the functional group, with a separation of 2 Å. Subsequently, the FChit/FP structures underwent optimization using the same theoretical approach as previously described. The optimized structures of the FChit/FP complexes are depicted in Fig. 3.

The adsorption energy (*E*_*ad*_), deformation energy (*E*_*def*_), and interaction energy (*E*_*int*_) of all studied Chit/Favi complexes were reported in Table 1. The calculated values of *E*_*ad*_ for all complexes are in the range of physisorption, which confirms the physical adsorption of the FP on Chit NPs through the weak van der Waals (vdW) interactions. Also, *E*_*ad*_ for adsorption of FP on pristine Chit is higher than the FChit NPs. In Fact, the adsorption energies of *N*-methyl- and Oxo-Chit/FP complexes are almost in the range of pristine chitosan, however, for *N*-acylate- and *O*-acetyl-Chit/FP complexes the *E*_*ad*_ values were reduced considerably. Noticeable mentioning that, for *N*-methyl-Chit/FP complex a strong chemical-bonding can be deduced from the interaction energy of − 95.91 kcal/mol. However, the high positive value of deformation energy (i.e., + 79.29 kcal/mol) reduced the *E*_*ad*_ to be in the range of physisorption. In other words, despite the strong chemical interaction in this case, the deformation of the *N*-methyl-Chit NP and FP molecule neutralizes the stability resulting from such strong interaction. A similar result can also be seen for the Oxo-Chit/FP complex, albeit with less intensity.

**Table 1:** Adsorption (E_ad_), deformation (E_def_), and interaction (E_int_) energies of the Chit/FP complexes calculated at B3LYP-D3(BJ)/6-311 + g(d,p) level of theory.

Complex	E_ad_	E_def_	E_i*n*t_
T1	− 12.14		
T2	− 8.776		
T3	− 8.447		
T4	− 6.685		
T5	− 4.574		
T6	− 4.574		
T7	− 10.467		
T8	− 12.185		
T9	− 11.043		
T10	− 10.136		
T11	− 16.702	1.44	− 18.15
T12	− 17.165	− 5.95	− 11.22
T13	− 11.765		
T14	− 14.902	3.41	− 18.32
N-acylate-Chit/FP	− 9.382	4.01	− 13.48
N-methyl-Chit/FP(water)	− 16.627	79.29	− 95.91
N-methyl-Chit/FP(gas)	− 18.452	67.61	− 86.06
O-acetyl-Chit/FP	− 12.589	0.8	− 13.38
Oxo-Chit/FP	− 16.488	3.53	− 20.01

All energy values are in kcal/mol. All calculations were done in aqueous environment using PCM model, except for the case of *N*-methyl-Chit/FP complex, which has been studied in the solution and gaseous states.

As can be seen, the *E*_*def*_ of *N*-methyl-Chit/FP complex is much more than the others, raising the question that perhaps this large deformation energy is may be due to the solvent effect. Therefore, FP, *N*-methyl-Chit, and their complex were optimized at B3LYP-D3(BJ)/6-311 + g(d,p) level in gaseous state, and the energy calculations were performed considering the BSSE correction in order to answer this question. The results were reported in Table 1, and the optimized structure of the complex were shown in Figure S2 of supplementary information. As can be seen, *E*_*def*_ in the gaseous state is + 67.61 kcal/mol and is 11.7 less than of the aqueous solution sate. Also, the *E*_*ad*_ and *E*_*int*_ of gaseous state are lower by − 1.83 and − 9.85 kcal/mol, respectively, than the aqueous state, which shows that the contribution of the water solvent to the structural deformation of the complex is about + 10 kcal/mol. In general, it can be said for *N*-methyl-Chit/FP complex that, the strong interaction between FP and *N*-methyl-Chit deforms the structure of FP molecule and causes the spatial twist around the CONH_2_ group of FP molecule. The deformation energy of FP molecule is about + 65.75 kcal/mol, and the instability resulting from this deformation reduces the absorption energy. It should also be mentioned that the amount of *N*-methyl-Chit deformation energy is much lower and is around + 1.85 kcal/mol.

**Figure 4:**
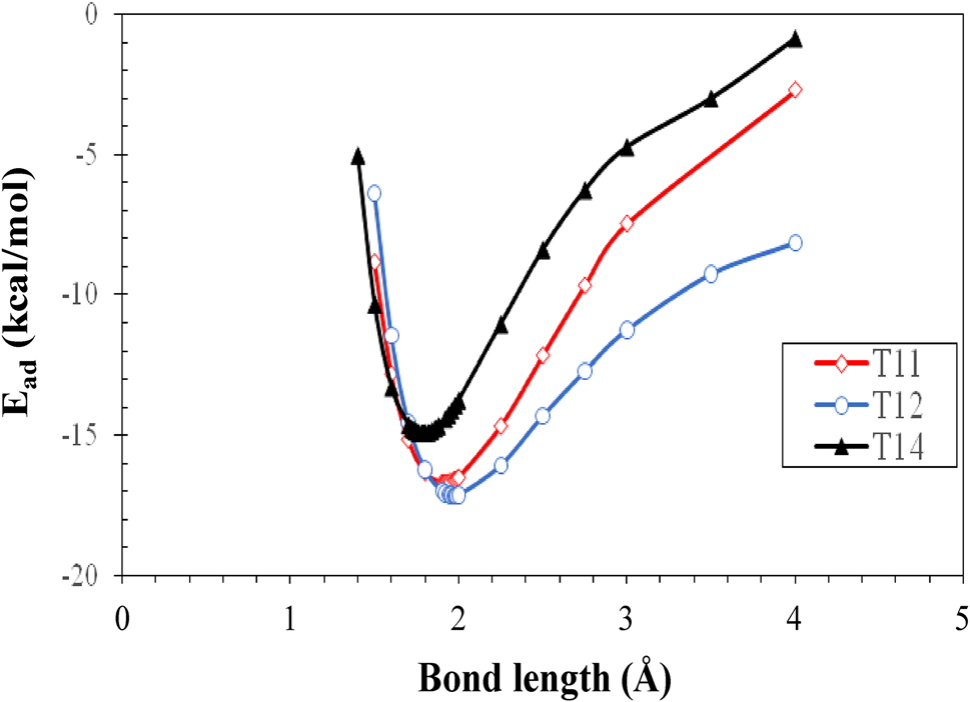
Adsorption energy (E_ad_) as a function of the distance between Chit and FP for pristine T11, T12, and T14 Chit/FP complexes.

**Figure 5:**
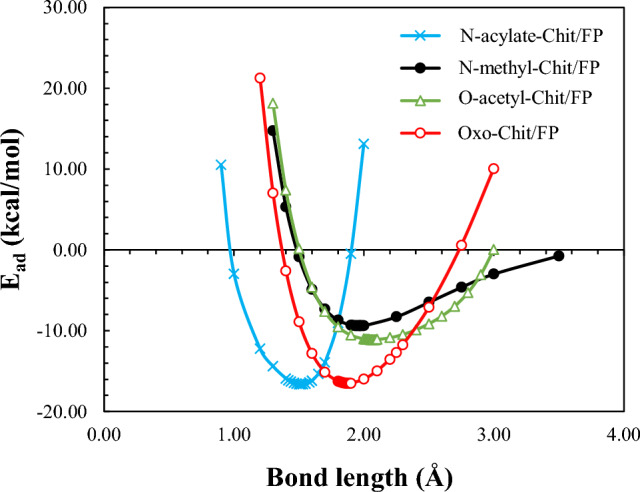
Adsorption energy (E_ad_) as a function of the distance between nanoparticle and FP for functionalized-Chit/FP complexes.

According to the adsorption energy values reported in Table 1, T11, T12, and T14 Chit/FP complex have the highest level of stability. For this reason, these structures along with all FChit/FP complexes were selected for further analysis. Also, to ensure that the obtained structures are really located at the minimum energy points, and therefore are stable, energy calculations were performed around the equilibrium distance for T11, T12, and T14 complexes of pristine-Chit/FP along with all FChit/FP complexes, and the results are shown in Figs. 4 and 5. These Figures confirms that all obtained structures are located in minima of the energy profile. Also, the lowest energy of each structure related to Figs. 4 and 5 are given in Table S1 of the supplementary information. It can be seen that these results are in complete agreement with the results of structure optimization which are reported in Table 1. In other words, it can be said that the optimized structures are all in a minimum energy point in terms of energy.

### Study the type and order of the possible interactions

#### NBO analysis

Using NBO analysis, the configuration results for electron donor–acceptor were obtained and the second-order perturbation estimation of the stabilization energy from such NBO interactions (E^2^) are presented in Table 2. In this table, the most important interactions in terms of stabilization energy as well as the electronic configuration for electron donor–acceptor of these interactions are reported. It can be observed from Table 2 that the strongest interactions (with the most negative E^2^) are for T11, T14, and *N*-methyl-Chit/FP complexes. In T11, the interactions are due to the donation of non-bonding lone pair electrons (LP) of N atom of Chit to the antibonding (BD*) type NBOs on H atom of FP as an acceptor. In T14 complex, the interactions are of LP(O, Chit) type as a donor and BD* type NBOs on H atom of FP as an acceptor. For N-methyl-Chit/FP complex, LP(O, FP) is a donor and BD* type NBOs on H atom of Chit is acceptor. Additionally, the presence of hydrogen bond interactions can be inferred in T11 between N (Chit) and H(FP); in T14 between O(Chit) and H(FP); and in *N*-methyl-Chit/FP complex between O(FP) and H(Chit). Furthermore, in T11 structure LP(N, Chit) interactions acts as a donor and BD*(N–H, FP) acts as an acceptor; in T14 LP(O, Chit) is a donor and BD*(N–H, FP) acts as an acceptor; in *N*-methyl-Chit/FP complex LP(O, FP) acts as a donor and BD*(N–H, Chit) is the acceptor. Additionally, in other structures such as T12, Oxo, *O*-acylated, and *N*-acylate-Chit/FP complexes weaker hydrogen bond interactions can be inferred. Based on the results of Table 2, non-bonding electron pairs are donated from LP to BD* in all bonds, and the most significant interactions are of hydrogen-binding type.

**Table 2:** The NBO analysis of the studied complexes.

Complex	Donor-NBO	Acceptor-NBO	E^2^ (kcal/mol)
T12	LP(O,FP)	BD*(O–H,chit)	3.23
	LP(O,FP)	BD*(O–H,Chit)	2.99
T11	LP(N,Chit)	BD*(N–H,FP)	19.11
	LP (N,FP)	BD*(C-H,Chit)	1.98
T14	LP (O,Chit)	BD*(N–H,FP)	10.96
	LP (O,FP)	BD*(O–H,Chit)	8.65
	LP (O,FP)	BD*(O–H,Chit)	4.81
*N*-acylate-Chit/FP	LP (O,Chit)	BD*(N–H,FP)	4.08
	LP (O,FP)	BD*(N–H,Chit)	2.42
	LP (O,FP)	BD*(N–H,Chit)	2.31
	LP (O,Chit)	BD*(N–H,FP)	1.52
*N*-methyl-Chit/FP	LP (O,FP)	BD*(N–H,Chit)	39.77
	LP (O,FP)	BD*(N–H,Chit)	12.22
	LP (N,FP)	BD*(N–H,Chit)	7.48
*O*-acetyl-Chit/FP	BD (O,Chit)	BD*(N–H,FP)	1.86
	LP (O,Chit)	BD*(N–H,FP)	1.4
	LP (O,Chit)	BD*(N–H,FP)	1.15
	LP (F,FP)	BD*(N–H,Chit)	1.11
Oxo-Chit/FP	LP (O,FP)	BD*(O–H,Chit)	5.58
	LP (O,Chit)	BD*(N–H,FP)	4.97
	LP (O,FP)	BD*(O–H,Chit)	4.67
	LP (O,Chit)	BD*(N–H,FP)	4.62
	LP (O,Chit)	BD*(N–H,FP)	4.38
	LP (O,FP)	BD*(O–H,Chit)	2.33

Bond length and Wiberg bond index (WBI) of the most stable possible interactions between FP and Chit nanoparticles are reported in Table 3. The natural atomic charges for the interacting atoms along with the charge difference between positive and negative charges (i.e., ∆Q = δ_NBO_^+^_−_δ_NBO_^−^) were also reported in this table. According to the WBI values, it can be said that most of the observed interactions between FP and chitosan NPs are of hydrogen-bond type. The results show that there is a direct relationship between the order of hydrogen bonds and the charge difference of two atoms, so that with the increase of charge, the order of hydrogen bonds increases and naturally their length decreases.

**Table 3:** Bond length (R_e_), Wiberg bond index (WBI), and natural charges (δ_NBO_) of Chit/FP complex.

Complex	Interaction	WBI	R_e_ (Å)	δ_NBO_^+^ (e)	δ_NBO_^−^ (e)	∆Q (e)
T12	O…H(–O)	0.0227	1.97	0.498(H)	− 0.698(O)	1.196
T11	H…N(–H)	0.0674	1.91	0.448(H)	− 0.857(N)	1.305
	N…H(–C)	0.0021	2.66	0.24(H)	− 0.439(N)	0.679
T14	H…O(–H)	0.0407	1.91	0.431(H)	− 0.790(O)	1.221
	O…H(–O)	0.0468	1.78	0.506(H)	− 0.740(O)	1.246
N-acylate-Chit/FP	H…O(–C)	0.0246	1.96	0.439(H)	− 0.717O)	1.156
	O…H(–N)	0.0168	2.11	0.426(H)	− 0.688(O)	1.114
N-methyl-Chit/FP	O…H(–N)	0.1469	1.51	0.489(H)	− 0.776(O)	1.265
	N…H(–N)	0.0281	2.09	0.416(H)	− 0.524(N)	0.94
O-acetyl-Chit/FP	H…O(–C)	0.0174	2.069	0.428(H)	− 0.638(O)	1.066
	F…H(–N)	0.0053	2.19	0.374(H)	− 0.352(F)	0.699
Oxo-Chit/FP	O…H(–O)	0.0283	1.87	0.504(H)	− 0.775(O)	1.279
	O…H(–O)	0.0217	1.96	0.488(H)	− 0.774(O)	1.262
	H…O(–C)	0.0287	1.93	0.144(H)	− 0.576(O)	0.72
	H…O(–C)	0.0216	2.05	0.433(H)	− 0.568(O)	1.001

Note that in Interaction column the left atom is related to FP and the right atoms belongs to chitosan nanoparticle. ∆Q = δ_NBO_^+^—δ_NBO_^−^.

#### QTAIM and NCI analysis

QTAIM analysis provide information on electron density (ρ(r)), Laplacian of electron density (∇^2^ρ(r)), electronic kinetic energy density (G(r)), electronic potential energy density (V(r)), and electronic Hamiltonian energy density (H(r) = G(r) + V(r)), which are reported in Table 4 for the selected bond critical points (BCPs) between Chit nanoparticles and FP molecules. Results for the other complexes can also be found Table S2 of the supplementary materials. According to the QTAIM data, electron density and Hamiltonian electronic energy density can be used to determine the strength of hydrogen bonding interactions in the bond critical points (BCP). This has been done in different studies by researchers to analyze the interaction between various compounds, including nanoscale or polymeric compounds with different drugs^55,56^.

**Table 4:** Values of the electron density (ρ(r)), Laplacian of electron density (∇^2^ρ(r)), electron kinetic energy density G(r), electron potential energy density V(r), electron Hamiltonian energy density H(r), and bond ellipticity (ε) for the selected critical points of Chit/FP complexes calculated at B3LYP-D3(BJ)/6–311 + g(d,p) theoretical level in the presence of water solvent.

Complex	interaction	ρ(r)	∇^2^ρ(r)	V(r)	G(r)	G(r)/|V(r)|	H(r)	ε
T11	H…NHC(R)	0.035	0.088	− 0.025	0.024	0.929	− 0.002	0.023
	N…HC(R)	0.011	0.032	− 0.006	0.007	1.21	0.001	0.059
	H…O(R)	0.011	0.034	− 0.006	0.007	1.164	0.001	0.074
T12	O…HOCC(R)	0.023	0.084	− 0.017	0.019	1.119	0.002	0.088
T14	O…HOCC(R)	0.035	0.125	− 0.031	0.031	1.001	0	0.024
	H…OHC(R)	0.028	0.094	− 0.021	0.022	1.051	0.001	0.02
*N*-acylate-Chit/FP	H…OCCH_3_NHC(R)	0.023	0.087	− 0.016	0.019	1.163	0.003	0.056
	O…HNC(R)COCH_3_	0.017	0.062	− 0.011	0.013	1.193	0.002	0.038
	C…HCH_2_CONHC(R)	0.011	0.039	− 0.006	0.008	1.255	0.002	1.363
*N*-methyl-Chit/FP	O…HNCH_3_C(R)	0.072	0.163	− 0.079	0.06	0.758	− 0.019	0.028
	O…HC(R)	0.015	0.051	− 0.01	0.011	1.167	0.002	0.063
	N…HNCH_3_C(R)	0.022	0.069	− 0.013	0.015	1.138	0.002	0.087
*O*-acetyl-Chit/FP	H…OCCH_3_OC(R)	0.018	0.067	− 0.012	0.014	1.204	0.002	0.01
	F…HNC(R)	0.013	0.049	− 0.009	0.011	1.161	0.001	0.04
Oxo-Chit/FP	O…HOC(R)	0.028	0.107	− 0.023	0.025	1.072	0.002	0.026
	H…OCHC(R)	0.024	0.092	− 0.018	0.021	1.14	0.003	0.018
	O…HOC(R)	0.023	0.085	− 0.017	0.019	1.129	0.002	0.07
	H…OCHC(R)	0.02	0.071	− 0.013	0.016	1.168	0.002	0.065

Electron density for the studied samples is obtained within the range of hundredths, indicating the weak interactions corresponding to the vdW interactions and hydrogen-bonds. The positive value of ∇^2^ρ(r) also further confirms the vdW interactions. This is also confirmed by the presence of G(r)/|V(r)| in the range of 1.0. In all selected interactions, the value of ε is very small, indicating a very stable hydrogen bond interaction. Only for the N-acylate-Chit/FP sample, ε value is higher than one, which is a sign of nonstable interaction in this case, and is in complete agreement with the low adsorption energy reported for N-acylate-Chit/FP complex. The proof of hydrogen bonding interactions can also be confirmed from around zero values of H(r). Also, for one of the interactions in *N*-methyl-Chit/FP complex, the electron density value close to 0.07 has been obtained, and G(r)/|V(r)| is almost 0.76, confirming that this interaction is a strong interaction of mixed covalent-hydrogen bond type. In Table 1 we confirmed the existence of such a strong interaction (with *E*_*int*_ = − 96 kcal/mol). According to the observations from Table 4, the electron density Laplacian is positive in all adsorption positions, indicating non-covalent bond formation.

**Figure 6:**
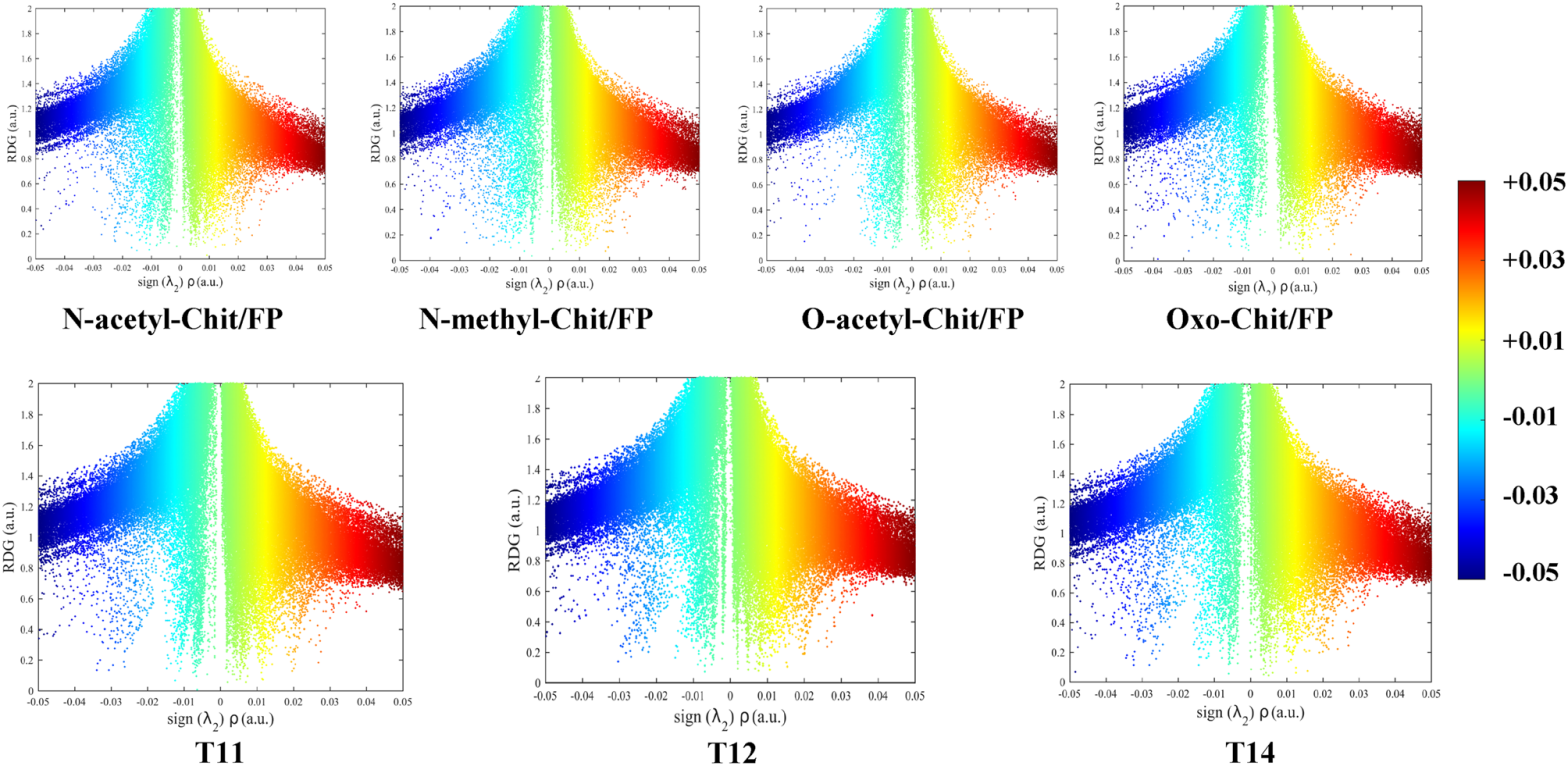
RDG plots of different Chit/FP complexes. The colorbar represents sign(λ)ρ(r) in atomic units.

The non-covalent interaction (NCI) analysis can also can be used to guarantee the QTAIM analysis. Reduced density gradient (RDG) plots of the studied complexes were presented in Fig. 6. In these plots, the positive region of the x-axis represents molecular repulsion, and the negative region represents molecular attraction. The regions from − 0.01to + 0.01 represent vdW interactions, and the regions from − 0.05- to − 0.01 is related to hydrogen bonds. According to the RDG plots, it can be observed that the highest density for the hydrogen bond region is obtained for the pure T12 complex. For the FChit/FP complexes, points in the hydrogen bond region are less dense, and therefore, the weaker hydrogen bonds are expected. As a general result, RDG plots, confirms the presence of hydrogen bonds in Chit/FP complexes and is in agreement with the results of previous analyses.

#### DOS, molecular orbitals, and conceptual DFT indices

**Figure 7:**
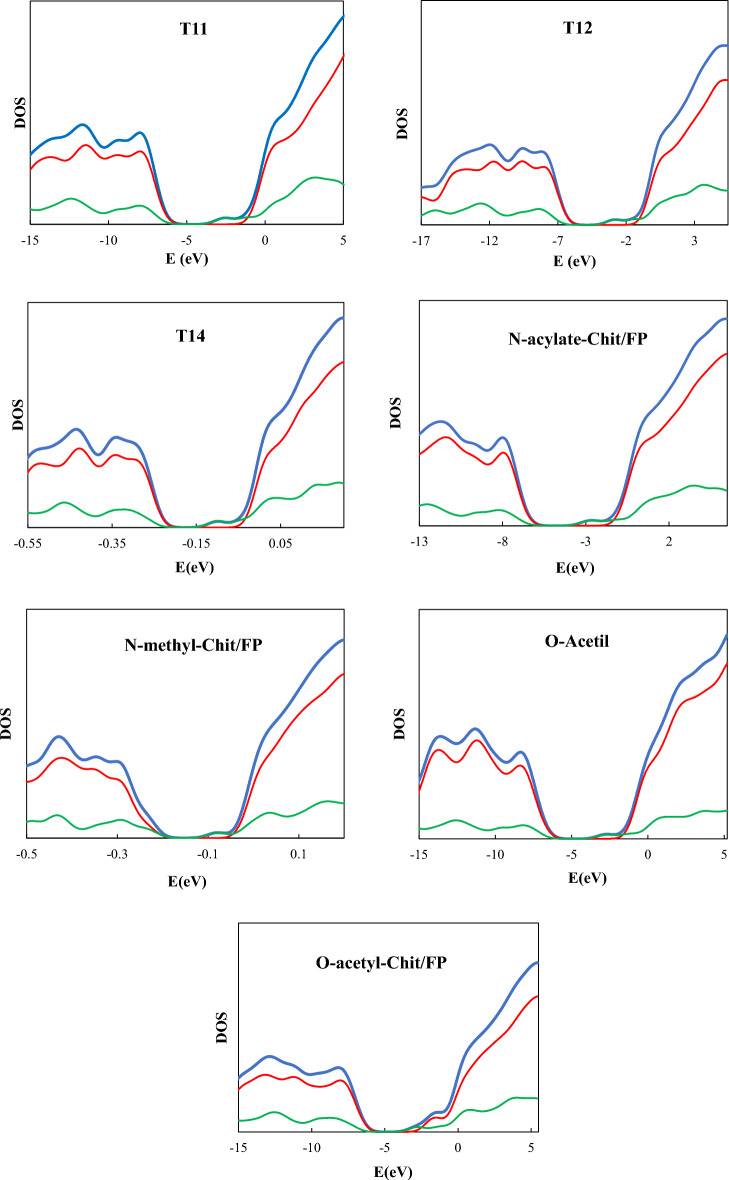
Density of states (DOS) spectra of the studied Chit/FP complexes. In all cases, blue, red, and green lines are the TDOS, PDOS(Chit), and PDOS(FP), respectively.

The splitting between the energy of frontier orbitals, i.e., HOMO and LUMO, as well as the electron density of state, DOS, can be used to study the charge and intramolecular energy exchange mechanism in a desired molecular system. Usually, by reducing the splitting between frontier orbitals, the amount of intramolecular electron exchange increases, which is related to the energy levels of filled/empty states and available states (related to DOS) of the molecule. In Fig. 7, DOS diagrams for pristine Chit/FP complexes are given.

From Fig. 7 for all Chit/FP complexes, the energy gap between HOMO and LUMO orbitals (HLG) was considerably decreased compared to the pristine Chitosan. This is due to the contribution of the LUMO orbitals of the FP in the TDOS in all cases other than Oxo-Chit/FP. In the case of Oxo-Chit/FP simultaneous participation of LUMO orbitals of FP molecule and Chit nanoparticles is seen. The physisorption of the FP on Chit can also be deduced from the insignificant changes in the intensity of the TDOSs. The substantial decrease of the HLG can be used in practical applications to detect the Chit/FP complex formation using the change in the electrical behavior (such as conductivity or resistance) of the aqueous solution of Chit and FP mixture.

The highest occupied molecular orbital (HOMO) and lowest unoccupied molecular orbital (LUMO) energy levels along with the conceptual-DFT indices were calculated to more understand the chemical behavior of the studied complexes. Considering that the atomic orbital expresses the highest probability (i.e., highest density) of the existence of electrons around the atoms, the study and investigation of the atomic orbital is of special importance. Chemical stability in molecules is measured by using the HOMO and LUMO energy levels and the conceptual-DFT indices introduced by Parr and Yang^50,57,58^. Some of the most widely used conceptual-DFT indices are chemical potential (μ), hardness (η), softness (S), and electrophilicity index (ω). Shortly, Chemical potential is directly related to the possibility of electron exchange of a molecule in the ground state with its surroundings. The chemical hardness measures the resistance of a molecule to exchange electrons with the surrounding, while softness has opposite meant (i.e., S = 1/η). Also, electrophilicity index is a criterion for measuring the stabilization resulting from the gaining of electrons by a molecule. The conceptual-DFT indices for the studied systems were reported in in Table 5.

**Table 5:** Conceptual-DFT indices for the studied systems.

System	ε_H_	ε_L_	HLG	µ	η	ω	S
FP	− 7.316	− 2.742	4.575	5.029	2.287	5.530	0.219
Chit	− 6.978	− 0.262	6.716	3.620	3.358	1.951	0.149
T11	− 6.917	− 2.605	4.312	4.761	2.165	5.265	0.232
T12	− 6.980	− 2.876	4.105	4.928	2.052	5.917	0.244
T14	− 6.963	− 2.736	4.227	4.849	2.114	5.563	0.237
*N*-acylate-Chit/FP	− 7.089	− 2.704	4.385	4.897	2.192	5.469	0.228
*N*-methyl-Chit/FP	− 6.198	− 2.166	4.032	4.182	2.016	4.338	0.248
*O*-acetyl-Chit/FP	− 7.095	− 2.737	4.357	4.916	2.179	5.546	0.229
Oxo-Chit/FP	− 6.964	− 2.808	4.156	4.886	2.078	5.744	0.241

ε_H_ and ε_L_ are energy levels of HOMO and LUMO orbitals, respectively, and HLG = ε_L_−ε_H_ is HOMO–LUMO gap.

All values are in eV.

**Figure 8:**
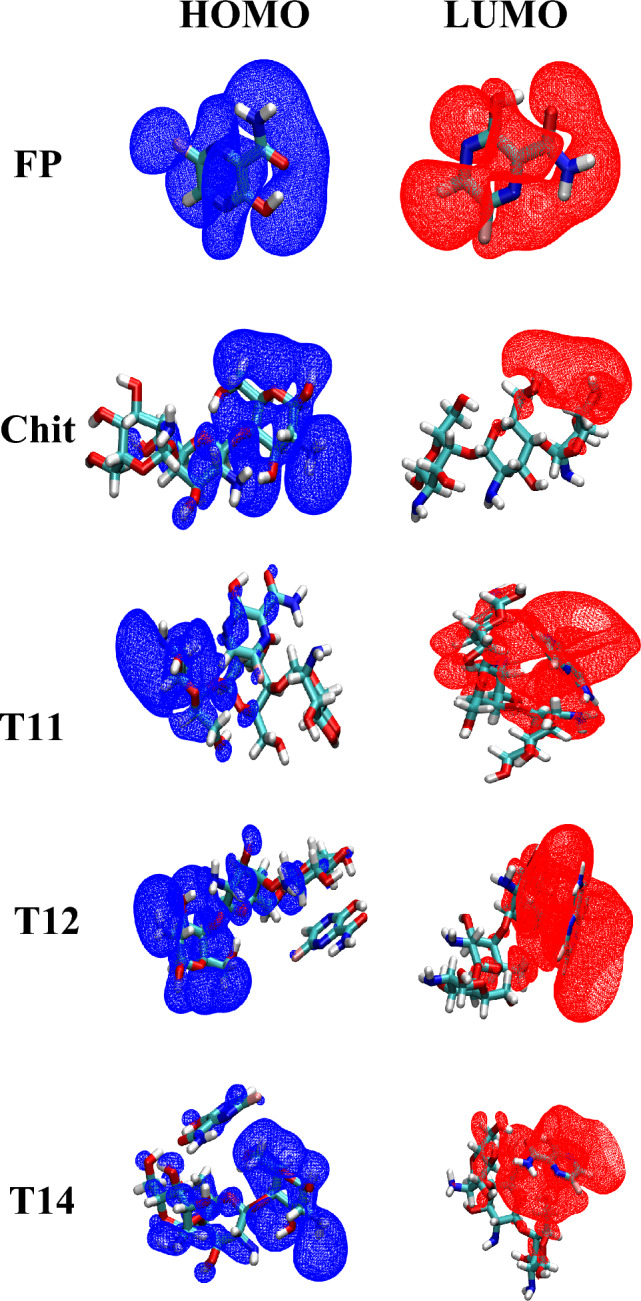
Frontier HOMO and LUMO molecular orbitals of FP molecule, Chit nanoparticles, as well as T11, T12, and T14 Chit/FP complexes.

**Figure 9:**
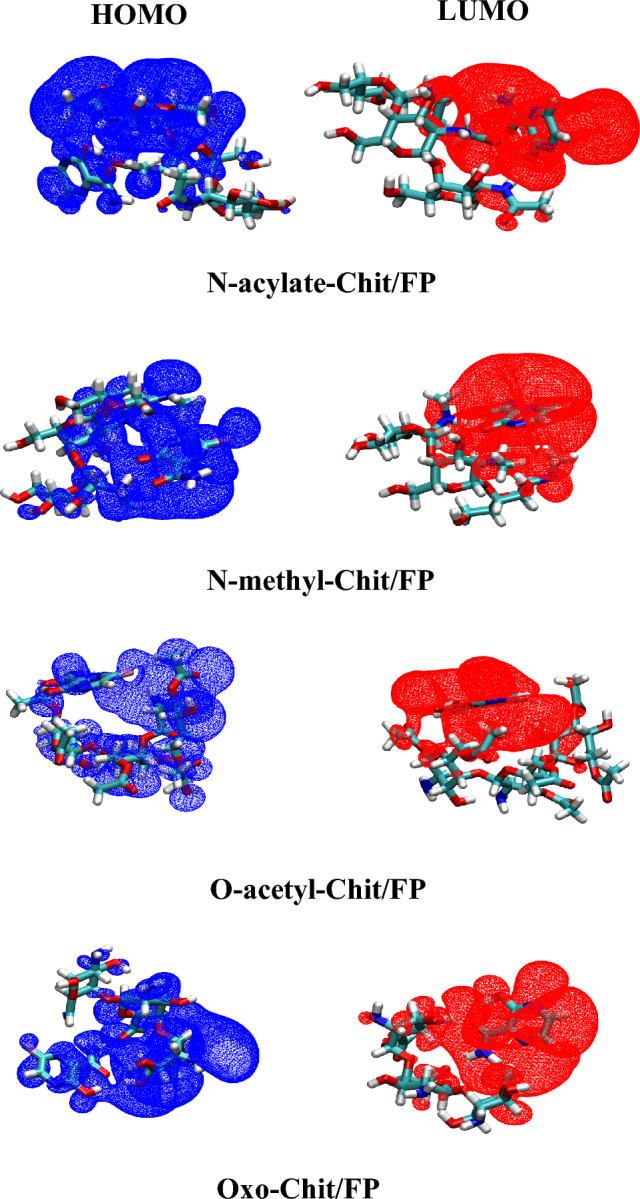
Frontier HOMO and LUMO molecular orbitals of functionalized Chit/FP complexes.

In all cases, it can be seen that the chemical potential of the complexes is in the average range of FP and Chit nanoparticles. The η values were decreased with the formation of Chit/FP complexes compared to the pristine Chit. The electrophilicity index of the complexes is close to the electrophilicity of pure FP, and the chemical softness of the complexes is higher than FP and polymer nanoparticles. Based on the principles of maximum hardness^59^ and minimum electrophilicity^60^, it can be say that the molecules tend to reach a state that has the highest hardness and the lowest electrophilicity. The decrease in hardness (η) in all cases shows that the resulting complex has become unstable and it is possible to easily release the FP drug by changing the environmental conditions. In other words, based on chemical hardness, it can be concluded that the resulting complex structures are suitable options in a targeted drug delivery. Also, the frontier HOMO and LUMO molecular orbitals for FP and studied Chit NPs along with complexes are shown in Figs. 8 and 9. From the comparison between the complexes and pure substnces shown this figure, it can be seen that the participation of the molecular orbitals of the HOMO and LUMO levels in the complexes is the cause of the interaction between FP and Chit NPs. Therefore, this figure confirms the existence of interaction between FP and chitosan nanoparticles.

It should also be mentioned that in this work, only the possibility of heterogeneous interactions between FP and Chit nanoparticle was investigated using ab-initio methods and using a very simple molecular model. It was shown that such interactions are mainly of the hydrogen-bonding type, and therefore Chit polymer can be a potential carrier of FP drug. There are also some experimental reports on the interaction of Chit with water molecules. In a study, Rao and coworkers^61^, by measuring some thermophysical properties, found that the hydrogen-bonding and complexation may the main reason of the Chit-solvent interactions in water + formic acid media. The polymer–solvent interaction parameter (χ) of Flory–Huggins theory can also be applied to estimate the energy of the interaction between the polymer segments and the solvent molecules. The value of χ = − 0.01 for the interaction of Chit with the 0.3 molar mixed solvent of acetic acid + water solution was reported by Safronov et al.^62^ They also concluded that, the solubility of the Chit in such mixed solvent is due to the interactions of hydrogen-bonding type between polar groups of Chit monomers and water molecules. In the real laboratory conditions, where the Chit and FP molecules are placed together in the solvent environment, naturally, the homogeneous interactions of FP-FP and Chit-Chit types are also existing, in addition to the homogenous FP-Chit interaction. Investigating such heterogenous interactions using classical and quantum computational methods is possible, which may be the subject of future studies.

## Conclusion

Considering the spread of the COVID-19 disease in recent years and the inappropriate performance of many drugs in the treatment of this disease, as well as the availability and cheapness of favipiravir (FP) drug, the purpose of the study was to use chitosan (Chit) nanoparticles in the drug-delivery of FP. Also, considering that pure chitosan is only soluble in limited acidic pHs (i.e., pHs < 6.5), some functionalized chitosan nanoparticles including *N*-acylate, *N*-methyl, *O*-acetyl, and Oxazoline functionalized chitosan nanoparticles, which are all soluble in water, were also studied along with the pristine chitosan. For this purpose, molecular studies using DFT theoretical methods were performed at B3LYP-D3(BJ)/6-311 + g(d,p) theoretical level. Initially, different complexes of Chit/FP were made and the resulting structures were optimized. Then, QTAIM, NBO, RDG, DOS, frontier molecular orbitals, and conceptual-DFT indices were used to investigate the nature of intermolecular interactions in the complexes. All analyzes showed that hydrogen-bonding type interactions are responsible for the formation of van der Waals complexes of FP with all studied Chit nanoparticles. In other words, the pristine and the functionalized chitosan NPs can all be used as a physical drug carrier for the targeted delivery of FP. Also, the results show that the functionalization of chitosan has not a significant effect on its drug-delivery ability, and if time and economic issues are preferable, pure chitosan nanoparticle is a suitable nano-carrier for FP drug delivery. Also, the functionalized Chit nanoparticles can be used to control drug release under specific pH conditions.

### Supplementary Information

Supplementary Information.

## Data Availability

All data generated or analyzed during this study are included in this published article.
