# Supplementary material for: Quantum DFT studies on the drug delivery of favipiravir using pristine and functionalized chitosan nanoparticles
Source: Sci Rep. 2023 Dec 11;13:21984. doi: 10.1038/s41598-023-49298-5 (PMC10713654; doi:10.1038/s41598-023-49298-5)
Supplement: Supplementary file 1 — Supplementary Information. [file 41598_2023_49298_MOESM1_ESM.pdf]

Supplementary Information

for

Quantum DFT Studies on the Drug Delivery of Favipiravir Using Pristine and Functionalized Chitosan Nanoparticles

Sheyda Ataei, Ebrahim Nemati-Kande*, Aidin Bahrami

Department of Physical Chemistry, Faculty of Chemistry, Urmia University, Urmia, Iran.

*E-mail address: nemati.ebrahim@gmail.com, e.nemati@urmia.ac.ir

Table S1. Minimum energy points of Figures 4 and 5. $E_{ad,m}$ is the minimum adsorption energy of Favipiravir on Chitosan, and R_m is the corresponding distance between the Favipiravir and Chitosan nanoparticles. All calculations were done at B3LYP-D3(BJ)/6-311+g(d,p) level of theory, and in the presence of water as solvent. The CPM solvent model was applied.

**Complex**	**R_m (Å)**	**$E_{ad,m}$ (kcal/mol)**
T11	1.91	-16.702
T12	1.98	-17.165
T14	1.78	-14.902
N-acylate-Chit/FP	1.97	-9.383
N-methyl-Chit/FP	1.52	-16.627
O-acetyl-Chit/FP	2.07	-11.083
Oxo-Chit/FP	1.88	-16.488

Table S2. Results of QTAIM analysis for all possible interactions at bond critical points (BCPs) between chitosan nanoparticles and Favipiravir drug. All calculations were done at B3LYP-D3(BJ)/6-311+g(d,p) level of theory, and in the presence of water as solvent. The CPM solvent model was applied. In all interactions the left atom belongs to the Favipiravir molecule and the right atoms are of the chitosan nanoparticles.

SComplex	interaction	ρ(r)	$\nabla^2\rho(r)$	V(r)	G(r)	|V(r)/G(r)|	H(r)	ε
T1	O...HN(R)	0.006	0.022	-0.004	0.005	1.238	0.001	1.28
	O...HC(R)	0.011	0.034	-0.006	0.007	1.16	0.001	0.025
	O...HC(R)	0.011	0.033	-0.006	0.007	1.172	0.001	0.024
	H...O(M)	0.027	0.1	-0.021	0.023	1.093	0.002	0.108
	N...HC(R)	0.006	0.02	-0.003	0.004	1.228	0.001	0.45
	N...O(R)	0.01	0.039	-0.007	0.008	1.219	0.001	1.008
T2	NH...OH(R)	0.026	0.096	-0.02	0.022	1.098	0.002	0.032
	N...HCO(R)	0.005	0.017	-0.003	0.004	1.234	0.001	0.86
	F...HCO(R)	0.007	0.026	-0.005	0.006	1.176	0.001	0.182
	F...HC(R)	0.007	0.025	-0.005	0.005	1.182	0.001	0.057
	F...HCO(R)	0.007	0.026	-0.005	0.006	1.169	0.001	0.106
T3	O...HN	0.016	0.057	-0.011	0.012	1.176	0.002	0.058
	NH...OH	0.036	0.123	-0.031	0.031	0.999	0	0.064
T4	N...HN	0.014	0.044	-0.007	0.009	1.261	0.002	0.06
	NH...OH	0.027	0.096	-0.021	0.022	1.079	0.002	0.049
T5	N...HCOC(R)	0.003	0.011	-0.002	0.002	1.238	0	1.08
	H...O(R)	0.017	0.053	-0.01	0.012	1.155	0.002	0.056
	F...HOC(R)	0.014	0.056	-0.01	0.012	1.187	0.002	0.041
T6	H...OH(R)	0.018	0.063	-0.011	0.014	1.2	0.002	0.096
	F...HN(R)	0.008	0.032	-0.006	0.007	1.211	0.001	0.046
T7	NH...OHC	0.023	0.078	-0.016	0.018	1.095	0.002	0.054
	O...HCO	0.009	0.03	-0.006	0.007	1.162	0.001	0.107
	C...HC(R)	0.005	0.017	-0.003	0.003	1.32	0.001	1.597
	O...OHC(R)	0.005	0.019	-0.003	0.004	1.195	0.001	1.132
	N...OHC(R)	0.005	0.016	-0.003	0.003	1.245	0.001	4.293
	C...OHC(R)	0.006	0.021	-0.003	0.004	1.261	0.001	2.059
T8	H...O(R)	0.022	0.085	-0.016	0.019	1.146	0.002	0.077
	N...HCC(R)	0.005	0.017	-0.003	0.004	1.262	0.001	0.553
	F...O(M)	0.002	0.013	-0.002	0.002	1.487	0.001	0.521
	F...HNC(R)	0.011	0.044	-0.008	0.01	1.175	0.001	0.049
	F...HC(R)	0.005	0.018	-0.003	0.004	1.235	0.001	0.052
	C...HC(R)	0.004	0.013	-0.002	0.003	1.335	0.001	0.97
	H...NHC(R)	0.007	0.023	-0.004	0.005	1.241	0.001	0.339
	N...HC(R)	0.008	0.025	-0.004	0.005	1.222	0.001	0.147
	N...HNC(R)	0.01	0.033	-0.006	0.007	1.222	0.001	0.22
	N...O(M)	0.003	0.011	-0.002	0.002	1.361	0.001	0.671

**T9**	H...OHC(R)	0.005	0.02	-0.003	0.004	1.265	0.001	0.838
	O...OHC(R)	0.004	0.016	-0.003	0.003	1.302	0.001	1.955
	O...HNC(R)	0.013	0.044	-0.009	0.01	1.142	0.001	0.111
	O...HC(R)	0.007	0.024	-0.004	0.005	1.176	0.001	0.789
	N...HNC(R)	0.008	0.024	-0.004	0.005	1.259	0.001	0.154
	C...HC(R)	0.005	0.013	-0.002	0.003	1.294	0.001	1.415
	F...OHC(R)	0.003	0.014	-0.002	0.003	1.359	0.001	0.121
	H...NHC(R)	0.018	0.051	-0.01	0.011	1.175	0.002	0.018
**T10**	N...HCC(R)	0.005	0.02	-0.003	0.004	1.374	0.001	0.632
	H...OHCC(R)	0.012	0.039	-0.007	0.009	1.155	0.001	0.046
	N... HCOHC(R)	0.009	0.028	-0.005	0.006	1.233	0.001	0.097
	F... HOCC(R)	0.01	0.039	-0.007	0.009	1.156	0.001	0.101
	O... OHC(R)	0.003	0.011	-0.002	0.002	1.315	0.001	0.443
	C...HC(R)	0.005	0.015	-0.002	0.003	1.265	0.001	1.058
	C...OHCC(R)	0.003	0.011	-0.002	0.002	1.351	0.001	0.056
	N...OHC(R)	0.007	0.023	-0.004	0.005	1.173	0.001	0.633
	O...HOC(R)	0.006	0.021	-0.004	0.005	1.179	0.001	0.361
**T13**	C...OHC(R)	0.009	0.032	-0.005	0.007	1.236	0.001	1.282
	N...HNC(R)	0.006	0.021	-0.004	0.004	1.228	0.001	0.243
	C...HC(R)	0.006	0.019	-0.003	0.004	1.255	0.001	3.067
	N...HCOHC(R)	0.008	0.023	-0.004	0.005	1.195	0.001	0.028
	H...O(M)	0.016	0.058	-0.01	0.012	1.195	0.002	0.065
	H...O(M)	0.006	0.02	-0.003	0.004	1.222	0.001	0.402

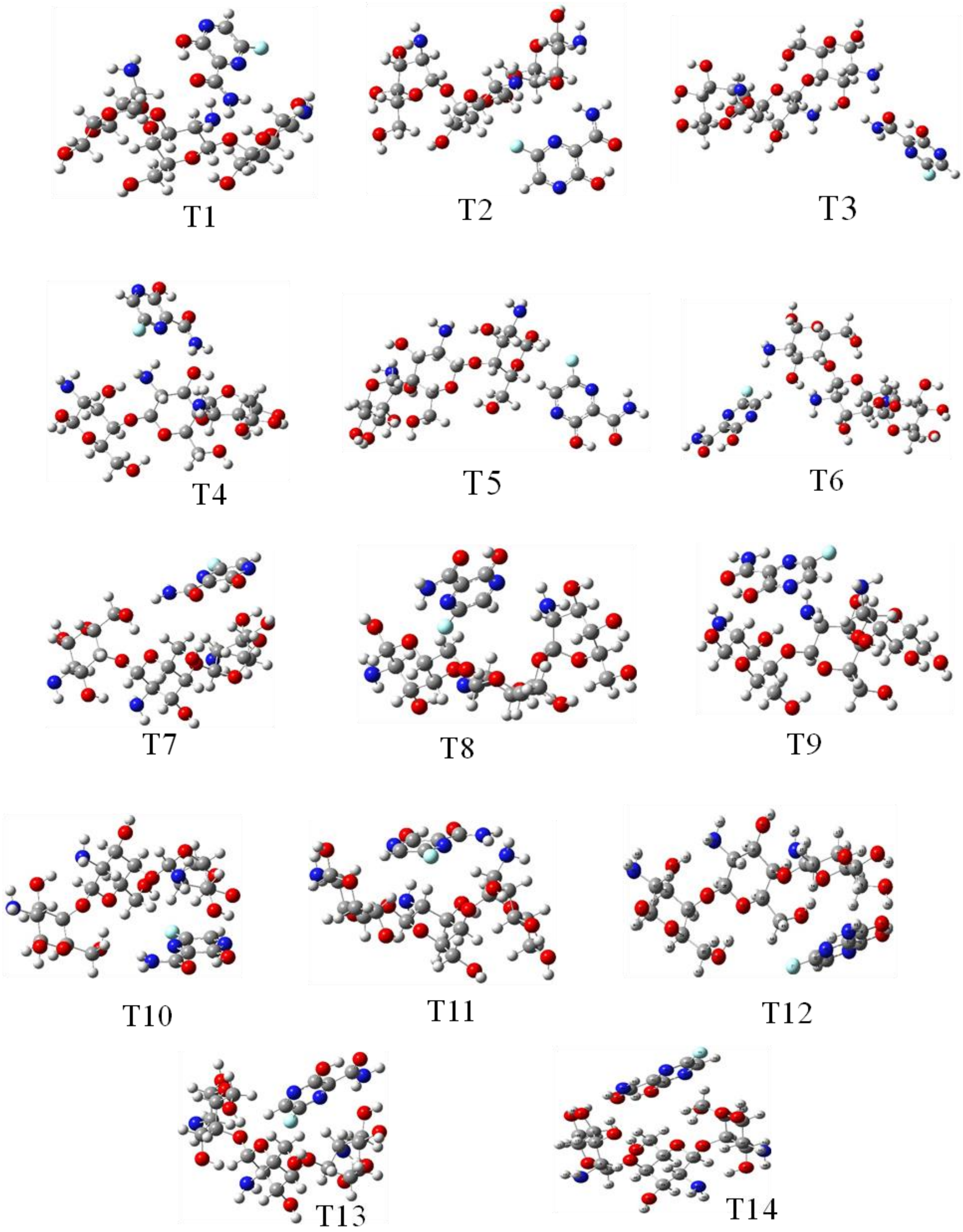

Figure S1. 14 optimized structure of the pristine Chit/FP complexes at B3LYP-D3(BJ)/6-311+g(d,p) level of theory, and in the presence of water solvent. The CPM solvent model was applied.

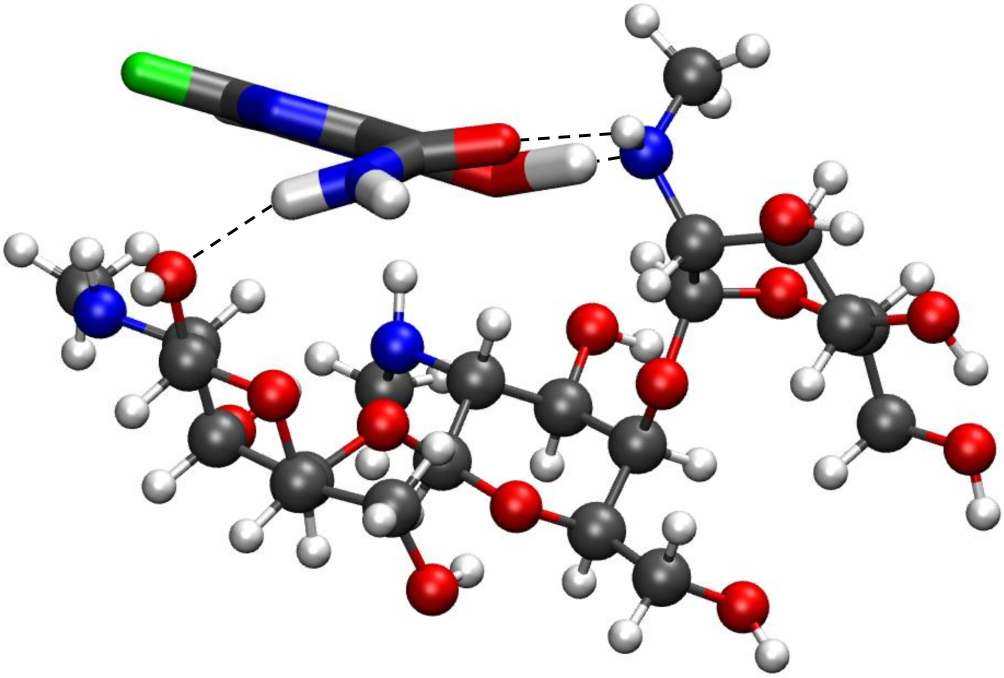

Figure S2. Optimized structure of the N-methyl-Chit/FP complex at B3LYP-D3(BJ)/6-311+g(d,p) level of theory in gaseous state.
